# Influence of postruminal casein infusion and exogenous glucagon-like peptide 2 administration on the jejunal mucosal transcriptome in cattle

**DOI:** 10.1371/journal.pone.0308983

**Published:** 2024-08-15

**Authors:** Ronald J. Trotta, Kendall C. Swanson, James L. Klotz, David L. Harmon

**Affiliations:** 1 Department of Animal and Food Sciences, University of Kentucky, Lexington, Kentucky, United States of America; 2 Department of Animal Science, North Dakota State University, Fargo, North Dakota, United States of America; 3 Forage-Animal Production Research Unit, United States Department of Agriculture, Agricultural Research Service, Lexington, Kentucky, United States of America; Zagazig University Faculty of Agriculture, EGYPT

## Abstract

We previously demonstrated that postruminal casein infusion and exogenous glucagon-like peptide 2 (GLP-2) administration independently stimulated growth and carbohydrase activity of the pancreas and jejunal mucosa in cattle. The objective of the current study was to profile the jejunal mucosal transcriptome of cattle using next-generation RNA sequencing in response to postruminal casein infusion and exogenous GLP-2. Twenty-four Holstein steers [250 ± 23.1 kg body weight (BW)] received a continuous abomasal infusion of 3.94 g raw corn starch/kg of BW combined with either 0 or 1.30 g casein/kg of BW for 7 d. Steers received subcutaneous injections at 0800 and 2000 h to provide either 0 or 100 μg GLP-2/kg of BW per day. At the end of the 7-d treatment period, steers were slaughtered for collection of the jejunal mucosa. Total RNA was extracted from jejunal mucosal tissue, strand-specific cDNA libraries were prepared, and RNA sequencing was conducted to generate 150-bp paired-end reads at a depth of 40 M reads per sample. Differentially expressed genes (DEG), KEGG pathway enrichment, and gene ontology enrichment were determined based on the FDR-corrected *P*-value (*padj*). Exogenous GLP-2 administration upregulated (*padj* < 0.05) 667 genes and downregulated 1,101 genes of the jejunal mucosa. Sphingolipid metabolism, bile secretion, adherens junction, and galactose metabolism were among the top KEGG pathways enriched with upregulated DEG (*padj* < 0.05) in response to exogenous GLP-2 administration. The top gene ontologies enriched with upregulated DEG (*padj* < 0.05) in response to exogenous GLP-2 administration included nutrient metabolic processes, brush border and bicellular tight junction assembly, and enzyme and transporter activities. Exogenous GLP-2 administration increased or tended to increase (*padj* < 0.10) brush border carbohydrase (*MGAM*, *LCT*, *TREH*), hexose transporter (*SLC5A1*, *SLC2A2*), and associated transcription factor (*HNF1*, *GATA4*, *KAT2B*) mRNA expression of the jejunal mucosa. Gene ontologies and KEGG pathways that were downregulated (*padj* < 0.05) in response to exogenous GLP-2 were related to genetic information processing. Postruminal casein infusion downregulated (*padj* < 0.05) 7 jejunal mucosal genes that collectively did not result in enriched KEGG pathways or gene ontologies. This study highlights some of the transcriptional mechanisms associated with increased growth, starch assimilation capacity, and barrier function of the jejunal mucosa in response to exogenous GLP-2 administration.

## Introduction

In North American beef cattle and dairy cattle production systems, grain-based diets containing moderate to large proportions of starch are typically fed to increase the net energy concentration of the diet to allow for more efficient growth and improved product quality. Depending on grain source and processing methods, up to 40% of dietary starch intake can escape ruminal fermentation and flow to the small intestine for potential enzymatic digestion [1]. Enzymatic digestion of starch in the small intestine can be energetically more efficient than ruminal fermentation of starch because absorption and oxidation of glucose provides more energy to the host than the production and oxidation of short-chain fatty acids [2]. However, small intestinal starch digestibility in ruminants is potentially limited by inadequate production of pancreatic and/or small intestinal carbohydrases [3, 4].

In prior studies, postruminal casein infusion increased pancreatic mass [5], pancreatic α-amylase mRNA expression, activity, and secretion [5–10], small intestinal starch digestibility [11–17], and net portal glucose absorption [18] in ruminants. Exogenous glucagon-like peptide 2 (GLP-2) has been shown to increase small intestinal mucosal growth by activating intracellular signaling pathways which promote cell proliferation and survival and inhibit cell proteolysis and apoptosis [19–21]. In pre-term parenterally-fed neonatal pigs, exogenous GLP-2 increased jejunal sucrase-isomaltase and maltase-glucoamylase mRNA expression, as well as, jejunal maltase and sucrase activity [22]. These prior studies suggest that postruminal casein infusion and exogenous GLP-2 administration would have independent effects on pancreatic and small intestinal growth and carbohydrase activities necessary for small intestinal starch hydrolysis.

With the goal of independently stimulating pancreatic α-amylase activity and jejunal α-glucosidase activity, we recently evaluated the effects of postruminal casein infusion and exogenous GLP-2 administration on growth, cellularity, and enzyme activity of the pancreas and small intestine in cattle [23]. In accordance with our hypotheses, postruminal casein infusion and exogenous GLP-2 administration differentially stimulated the growth and enzyme activities of the pancreas and small intestinal mucosa in cattle abomasally infused with starch [23]. Postruminal casein infusion increased pancreatic mass, protein content, and α-amylase activity [23]. Exogenous GLP-2 administration increased jejunal mass, mucosal mass, DNA content, and α-glucosidase activity (sum of maltase, isomaltase, and glucoamylase activity) [23]. To better understand the hydrolytic limitations of small intestinal starch digestion in cattle, deeper investigations into the molecular mechanisms associated with increased jejunal growth and α-glucosidase activity are needed.

Understanding epithelial cell function during periods of compromised nutrient utilization by the gastrointestinal tract, such as limitations in small intestinal starch hydrolysis, is a key opportunity for future research [24]. Although there have been several investigations focused on adaptation of the ruminal epithelium to high-starch diets [25–29], there is limited research examining functional measurements of the small intestinal epithelium combined with high-throughput molecular-based techniques to ascribe important phenotypes of ruminant animal production. It was hypothesized that RNA sequencing analyses would lead to the discovery of novel transcriptional mechanisms affected by postruminal casein infusion and exogenous GLP-2 administration. Therefore, the objective of this experiment was to evaluate the effects of postruminal casein infusion and exogenous GLP-2 administration on transcriptomic pathways and functions of the jejunal mucosa using next-generation RNA sequencing.

## Materials and methods

All surgical, animal care, and experimental protocols were approved by the University of Kentucky Animal Care and Use Committee (2020–3479).

### Animals, diet, and abomasal catheterization surgery

The experimental design was previously described by Trotta [23]. Briefly, 24 Holstein steers [initial body weight (BW) = 250 ± 23.1 kg; 10–12 months of age] were initially housed in individual pens (3 m × 3 m) in the Intensive Research Building of the University of Kentucky C. Oran Little Agricultural Research Center in Versailles, KY. Before surgery, steers were deprived of feed (36 h) and water (12 h), fitted with temporary jugular vein catheters (Mila International Inc., Florence, KY), and received a subcutaneous injection of ceftiofur (6.6 mg/kg of BW) and intravenous injection of flunixin meglumine (1 mg/kg of BW). Steers were sedated by intravenous injection of xylazine (0.088 mg/kg of BW) with ketamine hydrochloride (1.76 mg/kg of BW), intubated, and anesthesia was maintained with isoflurane in O_2_ using a ventilator in left lateral recumbency. The depth of anesthesia was monitored through observations of eye reflex and heart rate throughout the surgery. Steers were surgically fitted with infusion catheters in the abomasum that were constructed of Tygon tubing (6.35-mm internal diameter; Saint-Gobain North America, Malvern, PA) [30]. Flunixin meglumine was administered intravenously 24-h post-surgery and steers were monitored for abnormal feed intake, fecal consistency, behavior, and mobility for 7 d, and rectal temperatures were monitored for 3 d post-surgery.

Steers were limit-fed 5.65 ± 0.468 kg of an alfalfa hay cube-based diet (82.9% alfalfa hay cubes, 16.2% finely ground corn, 0.45% trace mineral premix, 0.45% vitamin premix; 40.7% neutral detergent fiber, 33.3% acid detergent fiber, 15.4% crude protein; 13.8% total starch, 1.32 Mcal/kg net energy for maintenance) on a dry matter (DM) basis. The diet was formulated to supply 1.33 times the net energy required for maintenance and exceed requirements for vitamins, minerals, ruminally degradable protein, and metabolizable protein for a steer gaining 0.46 kg/d [31]. Rations were provided twice daily at 0800 and 2000 h in equal portions. Steers were adapted to the basal diet for at least 7 d before the start of the treatment period.

### Experimental design

Steers were transferred to metabolism stalls (1.2 m × 2.4 m) for the 7-d treatment period. Due to a limited number of stalls and infusion apparatus, steers were stratified by BW into 6 replicate blocks. Steers were then randomly assigned to one of four treatments within each replicate block. The experimental design was a randomized complete block design with a 2 × 2 factorial arrangement of treatments. All steers were abomasally infused with 3.94 ± 0.245 g raw corn starch · kg BW^-1^ · d^-1^. Along with raw corn starch, steers were abomasally infused with either water or 10% (wt:wt) sodium caseinate solution to provide 0 or 1.30 ± 0.299 g sodium caseinate · kg BW^-1^ · d^-1^. Additionally, steers received subcutaneous injection treatments in two equal portions daily at 0800 and 2000 h. Subcutaneous injection treatments were either vehicle (5 g/L bovine serum albumin (BSA) diluted in 9 g/L NaCl) or 100 μg GLP-2 in vehicle · kg BW^-1^ · d^-1^. This resulted in four treatments: 1) abomasal water + vehicle injection (control), 2) abomasal water + GLP-2 injection (GLP-2), 3) abomasal 10% sodium caseinate solution + vehicle injection (casein), and 4) abomasal 10% sodium caseinate solution + GLP-2 injection (casein + GLP-2). All steers received abomasal infusion and subcutaneous injection treatments for 7 d. The 7-d treatment period was chosen because postruminal casein infusion has been shown to increase small intestinal starch disappearance within 6 d [12] and acute (1 d) or chronic (10 d) exposure to GLP-2 at the same dosage level achieved pharmacological plasma concentrations of GLP-2 in cattle [32].

### Glucagon-like peptide 2 preparation and administration

Glucagon-like peptide 2 was synthesized (Alan Scientific Inc., Gaithersburg, MD) based on the native bovine sequence [33, 34]. The purity (96%) was quantified by the manufacturer using high-performance liquid chromatography. The GLP-2 solution was prepared to a final concentration of 5 mg/mL by dissolving GLP-2 in 5 g/L BSA in 9 g/L NaCl solution. Aliquots were frozen in 10 mL screw-cap tubes at -20°C until use. Intestinotrophic effects of GLP-2 are greater when GLP-2 was administered subcutaneously compared with intramuscular or intraperitoneal injections [35]. Therefore, treatments were administered subcutaneously in the neck region in front of the shoulder. Left and right lateral administration sites were alternated for morning and evening injections, respectively.

### Nutrient infusion preparation and techniques

Due to large inter- and intra-animal variability in postruminal starch flows when high-starch diets are fed to cattle [3, 36], postruminal starch flow was controlled via continuous abomasal infusion of starch. Raw corn starch (Clinton 185 Corn Starch; Archer Daniels Midland Company, Decatur, IL) was chosen as the starch source to facilitate the greatest limitations in small intestinal starch digestibility, as the action of pancreatic α-amylase and small intestinal α-glucosidases are required for complete hydrolysis to glucose. The chosen amount of infused raw corn starch was predicted to limit the extent of small intestinal starch disappearance [37] and mimic natural amounts of postruminal starch flow in cattle fed high-grain diets [38]. Additionally, infusion of 986 g of raw corn starch per day equates to 41.1 g/h, which does not cause diarrhea and appreciable amounts (10 to 15 g/h) of infused carbohydrate were expected to pass the ileocecal junction [39]. Sodium caseinate (AMCO Proteins, Burlington, NJ) was chosen as the protein source because of its relatively high solubility in water and because approximately 70–85% of luminal amino acids are transported across the apical membrane as small peptides [40]. The amount of casein was chosen relative to the amount of corn starch infused (1 part casein: 3 parts corn starch) and because previous experiments have demonstrated increased small intestinal starch disappearance [11, 15] and pancreatic α-amylase secretion [7] at similar levels.

Two containers of infusate suspensions per steer were prepared daily, immediately before infusion, for use over 12-h intervals. Infusate suspensions were maintained through continuous stirring via a stir bar with stir plate. Infusate suspensions (3.26 ± 0.109 kg) were prepared using the appropriate amount of raw corn starch, 5% (wt:wt) CrEDTA solution as an indigestible flow marker [41], and tap water every 12 h for each steer. A 10% (wt:wt) solution of sodium caseinate [42] was prepared with warm tap water in an insulated kettle (LEC-40; Legion Industries Inc., Waynesville, GA) using an industrial electric mixer (CDP3330; Baldor Electric Co., Ft. Smith, AR). The solution was mixed for at least 2 h and then was allowed to rest for 8 h to allow for air bubbles to escape [43]. The final solution was stored in 18.9-L buckets at -20°C until use. For casein treatments, the sodium caseinate solution replaced tap water in the infusate suspension. Corn starch and casein solute concentrations and abomasal infusion rates were similar to those reported by Kreikemeier [39] and Richards [15]. Abomasal infusion treatments were continuously infused (271 ± 9.08 g infusate/h) through Tygon tubing (3.18-mm internal diameter) using a multi-channel peristaltic pump (205U/CA; Watson-Marlow, Falmouth, United Kingdom). Infusion tubing was inserted into abomasal infusion catheters and secured with hose clamps. The amount infused was determined by the weight of the residual infusate after each 12-h period. The infusate containers were placed on top of a portable shelf (> 2 m height) to aid in the pumping of the suspension. Measures of fecal flow and postruminal starch disappearance from steers used in the current study were previously reported [23].

### Tissue collection

Upon completion of the 7-d infusion period, the infusion tubing was disconnected from abomasal infusion catheters and steers were transported to the University of Kentucky Meats Laboratory within 1 h. Steers were humanely stunned via captive bolt and exsanguinated. After slaughter, the viscera were rapidly removed and separated for individual weights and subsample collection. The pyloric and ileocecal junctions were cut to separate the small intestine from the abomasum and cecum, respectively, and the mesentery was cut to separate the entire small intestine from the viscera. The small intestine was measured in length by looping the intestine between pegs at either end of a 2.43-m board [44]. The small intestine was separated into the duodenum (0.1 to 1.1 m caudal to the pyloric sphincter), jejunum (proximal half of non-duodenal small intestine), and ileum (distal half of non-duodenal small intestine) [45]. After the small intestinal length was measured, digesta was gently removed from the lumen of the small intestine. Each intestinal section (duodenum, jejunum, ileum) was weighed and sampled (1 m) from the midpoint. Each 1-m segment was cut into three equidistant subsamples, everted, and rinsed in ice-cold saline. The first subsample was cut laterally, scraped with a glass microscope slide, and 200 mg of mucosa was weighed and preserved with RNAlater stabilization solution (Thermo Scientific Inc., Waltham, MA) in RNase-free tubes. Tubes were initially stored at 4°C for 24 h to allow for tissue penetration and then were transferred to -80°C storage, as recommended by the manufacturer. The second subsample was scraped as described previously and the mucosa was flash-frozen in liquid nitrogen in an aluminum pouch and stored at -80°C for cellularity and enzymatic analyses. The third subsample was weighed, mucosa scraped from the entire section, and then the isolated mucosa was weighed. Measures of small intestinal length and mass, cellularity, and enzyme activity were previously published [23].

### RNA isolation, library preparation, and sequencing

The jejunal mucosa was selected for RNA isolation and sequencing because small intestinal α-glucosidase activity (maltase, isomaltase, glucoamylase), Na^+^-dependent glucose uptake activity, and mRNA expression of intestinal glucose transporters (*SLC5A1* and *SLC2A2*) is greatest in the jejunum of cattle [44, 46, 47]. Additionally, relative mRNA expression of the GLP-2 receptor (*GLP2R*) is greater in the jejunum than other gastrointestinal tissues [48]. Approximately 200 mg of jejunal mucosal tissue was shipped for RNA extraction, library preparation, and RNA sequencing by Novogene Corporation Inc. (Sacramento, CA). Total RNA was extracted from jejunal mucosal tissue using TRIzol reagent (Thermo Fisher Scientific Inc., Waltham, MA). RNA concentration (1,003 ± 863 ng/μL) was measured at 260 nm using a microvolume UV-VIS spectrophotometer (NanoDrop; Thermo Fisher Scientific Inc., Waltham, MA) and purity was assessed using the 260/280 nm ratio (range = 1.8 to 2.2) for protein contamination and 260/230 nm ratio (≥ 1.8) for nucleic acid contamination. Samples were assessed for RNA integrity (mean ± standard deviation = 6.59 ± 1.30; range = 4.2 to 9; median = 7) using the RNA 6000 Nano Kit (Agilent Technologies Inc., Santa Clara, CA) and automated electrophoresis (2100 Bioanalyzer Instrument; Agilent Technologies Inc., Santa Clara, CA). One sample in the control treatment was not prepared for RNA sequencing due to low RNA integrity (RIN < 4).

Strand-specific cDNA library preparation was conducted by Novogene Corporation Inc. (Sacramento, CA). Messenger RNA was isolated from total RNA using poly-T oligo-attached magnetic beads. Fragmentation was facilitated using divalent cations under elevated temperature in First Strand Synthesis Reaction Buffer (5X). First-strand cDNA was synthesized using random hexamer primer and Moloney murine leukemia virus reverse transcriptase and second-strand cDNA was synthesized using DNA polymerase I and RNAse H. Remaining overhangs were converted to blunt ends using exonuclease and polymerase activities. Three-prime ends of DNA fragments were adenylated and adaptors with hairpin loop structures were ligated for hybridization preparation. Library fragments were purified with AMPure XP System (Beckman Coulter Inc., Brea, CA) and 370–420 bp cDNA fragments were selected for PCR amplification. Polymerase chain reaction products were purified and assessed with the 2100 Bioanalyzer Instrument (Agilent Technologies Inc.). Strand-specific RNA sequencing was conducted NovaSeq 6000 Sequencing System (Illumina Inc., San Diego, CA) to generate 150-bp paired-end reads at an average depth of 55,666,406 reads per sample.

### Bioinformatic and statistical analyses

Raw reads were filtered to remove sequencing adaptors, low-complexity reads, and reads containing low-quality bases using fastp software [49]. Quality control scores (Q20 = 96.8 ± 0.370%; Q30 = 91.41 ± 0.831%) and GC content (47.9 ± 1.25%) were calculated. Paired-end clean reads were aligned to the *Bos taurus* reference genome (ARS-UCD1.2) using HISAT 2 (version 2.0.5) [50]. Mapped reads of each sample were assembled using StringTie (version 1.3.3b) [51] in a reference-based approach. There was an average of 40,644,458 uniquely mapped reads per sample (73.0% of total reads). Reads per gene were counted using featureCounts (version 1.5.0-p3) [52]. Quantification of each gene was calculated as the number of fragments per kilobase of transcript sequence per million base pairs sequenced (FPKM). Visualization of box plots, violin plots, and FPKM density plots confirmed that gene expression was distributed equally across each steer and that the FPKM density conformed to a negative binomial distribution. Differential gene expression (DEG) analysis was quantified using the DESeq2 R-package (version 1.20) [53].

The DEG analysis used the negative binomial generalized linear model to fit gene expression level as a negative binomial distribution and Wald statistics to perform hypothesis testing. Multiple testing adjustment of the *P*-values was performed using the Benjamini-Hochberg procedure for controlling the false discovery rate (FDR). Genes were identified as differentially expressed if the FDR-adjusted *P*-value (*padj*) was ≤ 0.05. Gene ontology and KEGG pathway enrichment analyses of DEGs were conducted using the clusterProfiler R-package [54]. Enrichment analyses were considered significant if the FDR-adjusted *P*-value was ≤ 0.05. Tendencies were declared when 0.05 < FDR-adjusted *P* ≤ 0.10. Changes in gene expression were classified as upregulated or downregulated based on the sign of the log2 fold change.

Initially, pairwise comparisons between each of the four treatments were assessed. However, casein did not generate DEG that collectively affected KEGG pathway or gene ontology enrichment compared with control. In addition, there were minimal effects that resembled a casein × GLP-2 treatment interaction when GLP-2 vs. casein + GLP-2 treatments were compared. Therefore, all subsequent data analyses were conducted to compare the main effects of postruminal casein infusion (n = 11 for water, n = 12 for casein) and exogenous GLP-2 administration (n = 11 for BSA, n = 12 for GLP-2).

## Results

A complete description of differentially expressed genes, KEGG pathways, and gene ontologies can be found in supporting information (S1–S8 Files).

### Summary of results

A summary of the effects of exogenous GLP-2 administration on differentially expressed genes, KEGG pathways, and gene ontologies is presented in Table 1. Exogenous GLP-2 administration upregulated (*padj* < 0.05) 667 DEGs, 26 KEGG pathways, 198 biological processes, 56 cellular components, and 60 molecular functions. Exogenous GLP-2 downregulated (*padj* < 0.05) 1,101 DEGs, 14 KEGG pathways, 270 biological processes, 105 cellular components, and 46 molecular functions. Postruminal casein infusion did not result (*padj* > 0.05) in any enriched KEGG pathways or gene ontologies of the jejunal mucosa.

**Table 1:** Summary of the effects of exogenous GLP-2 administration on the number of differentially expressed genes, KEGG pathways, and gene ontologies of the jejunal mucosa.

Analysis	Upregulated	Downregulated
Differentially expressed genes	667	1,101
KEGG pathways	26	14
Gene ontologies		
Biological processes	198	270
Cellular components	56	105
Molecular function	60	46

### Differentially expressed genes

**Fig 1:**
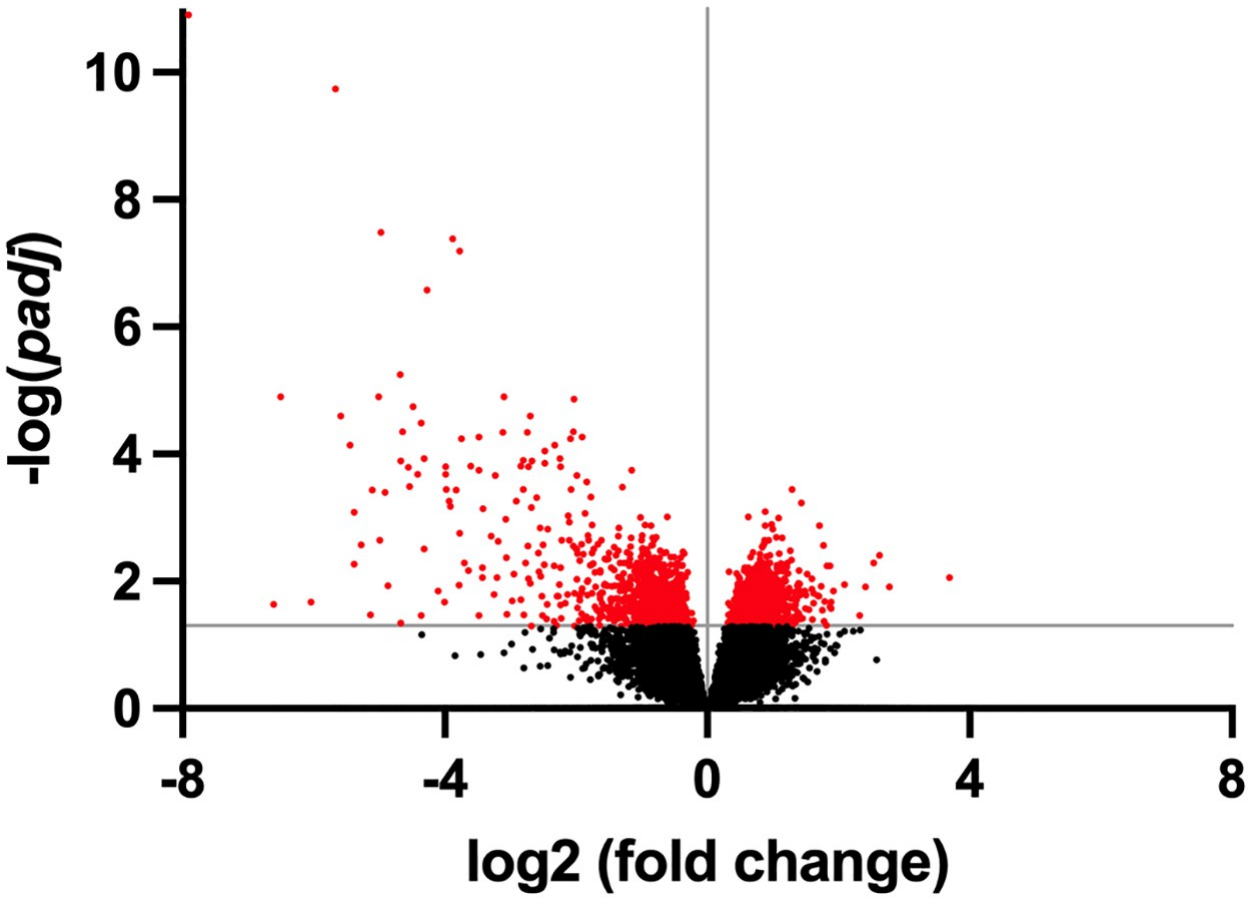
Volcano plot of jejunal mucosal genes influenced by exogenous GLP-2 administration. Data points highlighted in red were differentially expressed (*padj* < 0.05).

Exogenous GLP-2 administration resulted in 1,768 DEGs (*padj* < 0.05) with 667 upregulated genes and 1,101 downregulated genes (Fig 1). Postruminal casein infusion resulted in 7 DEGs (*padj* < 0.05) with 0 upregulated genes and 7 downregulated genes in the jejunal mucosa. Transcripts of the jejunal mucosa that were downregulated (*padj* < 0.05) with postruminal casein infusion include *ENSBTAG00000047783* (log2 fold change = −4.00), *ENSBTAG00000040367* (log2 fold change = −6.07), *ENSBTAG00000047029* (log2 fold change = −1.13), *CCR7* (C-C motif chemokine receptor 7; log2 fold change = −2.41), *SFRP5* (secreted frizzled related protein 5; log2 fold change = −4.11), *FCER2* (FC epsilon receptor II; log2 fold change = −3.48), and *C1QTNF2* (C1q and TNF related 2; log2 fold change = −5.41) (S4 File).

### Gene ontologies

**Fig 2:**
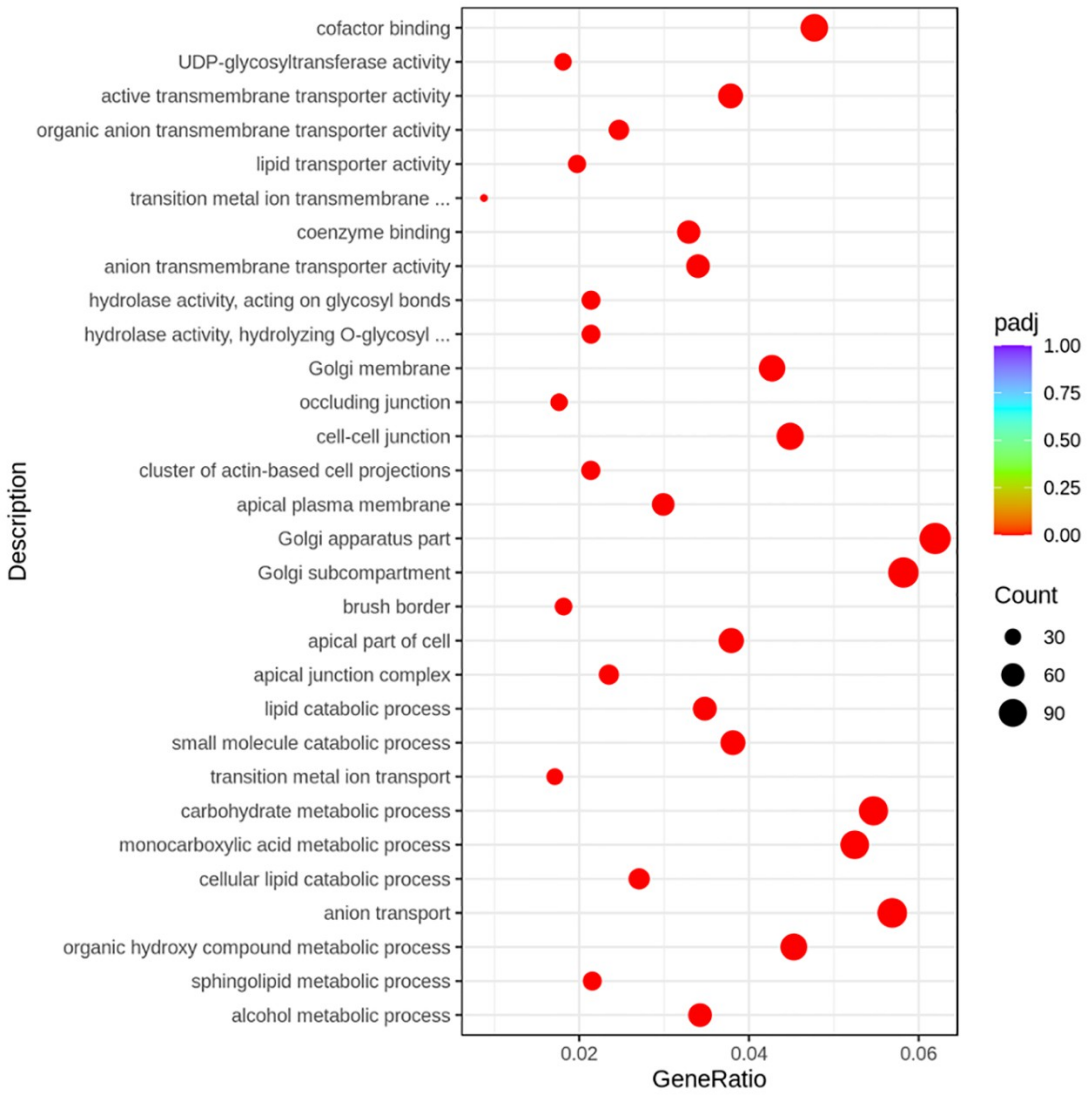
Top enriched gene ontology terms of the jejunal mucosa that were upregulated with exogenous GLP-2 administration. GeneRatio is the ratio of upregulated DEGs to all genes in a gene ontology. *Padj* is the probability value for hypergeometric test adjusted for Benjamini-Hochberg FDR correction.

The top 10 biological processes that were upregulated (*padj* < 0.05) with exogenous GLP-2 administration were the alcohol metabolic process, sphingolipid metabolic process, organic hydroxy compound metabolic process, anion transport, cellular lipid catabolic process, monocarboxylic acid metabolic process, carbohydrate metabolic process, transition metal ion transport, small molecule catabolic process, and lipid catabolic process (Fig 2). The top 10 cellular components that were upregulated (*padj* < 0.05) with exogenous GLP-2 administration were apical junction complex, apical part of the cell, brush border, Golgi subcompartment, Golgi apparatus part, apical plasma membrane, the cluster of actin-based cell projections, cell-cell junction, occluding junction, and Golgi membrane. The top 10 molecular functions that were upregulated (*padj* < 0.05) with exogenous GLP-2 administration were hydrolase activity (hydrolyzing *O*-glycosyl compounds), hydrolase activity (hydrolyzing glycosyl bonds), anion transmembrane transporter activity, coenzyme binding, transition metal ion transmembrane transporter activity, lipid transporter activity, organic anion transmembrane transporter activity, active transmembrane transporter activity, UDP-glycosyltransferase activity, and cofactor binding.

**Fig 3:**
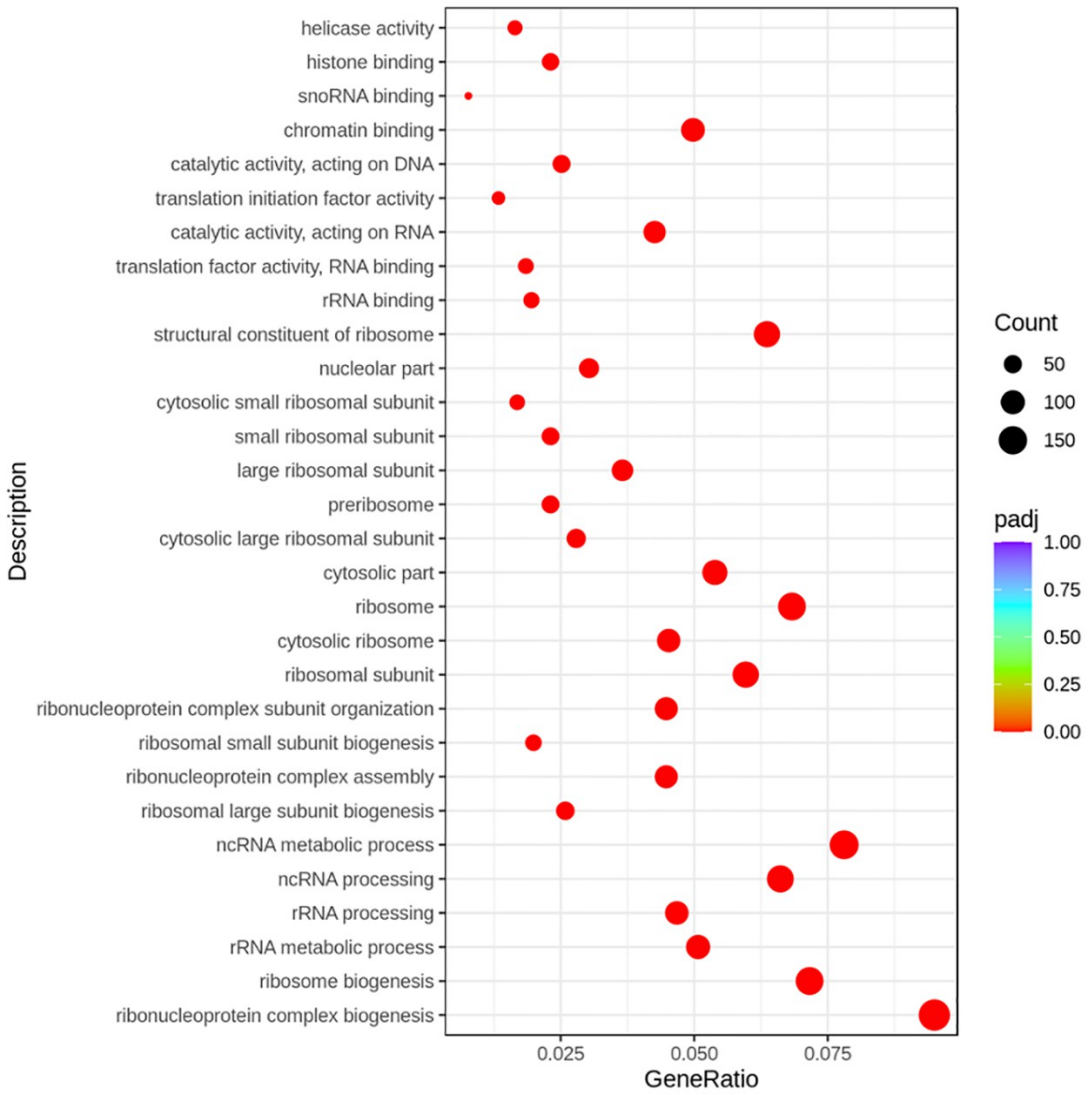
Top enriched gene ontology terms of the jejunal mucosa that were downregulated with exogenous GLP-2 administration. GeneRatio is the ratio of upregulated DEGs to all genes in a gene ontology. *Padj* is the probability value for hypergeometric test adjusted for Benjamini-Hochberg FDR correction.

The top 10 biological processes that were downregulated (*padj* < 0.05) with exogenous GLP-2 administration were ribonucleoprotein complex biogenesis, ribosome biogenesis, rRNA metabolic process, rRNA processing, ncRNA processing, ribosomal large subunit biogenesis, ribonucleoprotein complex assembly, ribosomal small subunit biogenesis, and ribonucleoprotein complex subunit organization (Fig 3). The top 10 cellular components that were downregulated (*padj* < 0.05) with exogenous GLP-2 administration were ribosomal subunit, cytosolic ribosome, ribosome, cytosolic part, cytosolic large ribosomal subunit, preribosome, large ribosomal subunit, cytosolic small ribosomal subunit, and nucleolar part. The top 10 molecular functions that were downregulated (*padj* < 0.05) with exogenous GLP-2 administration were the structural constituent of ribosome, rRNA binding, translation factor activity (RNA binding), catalytic activity (acting on RNA), translation initiation factor activity, catalytic activity (acting on DNA), chromatin binding, snoRNA binding, histone binding, and helicase activity.

### KEGG pathways

The KEGG pathways that were upregulated (*padj* < 0.05) with exogenous GLP-2 administration were sphingolipid metabolism, adherens junction, bile secretion, galactose metabolism, vitamin digestion and absorption, mineral absorption, peroxisome, carbon metabolism, tight junction, other glycan degradation, starch and sucrose metabolism, retinol metabolism, lysosome, insulin resistance, ascorbate and aldarate metabolism, *N*-glycan biosynthesis, valine leucine and isoleucine degradation, glycerolipid metabolism, PPAR signaling pathway, and lysine degradation, protein digestion and absorption, AMPK signaling pathway, fat digestion and absorption, propanoate metabolism, gastric cancer, and various types of *N*-glycan biosynthesis (Table 2).

**Table 2:** Enriched KEGG pathways of the jejunal mucosa that were upregulated with exogenous GLP-2 administration^1^.

KEGG ID	Description	GeneRatio	BgRatio	*pvalue*	*padj*
bta00600	Sphingolipid metabolism	25/1,021	48/7,748	<0.0001	<0.0001
bta04520	Adherens junction	27/1,021	75/7,748	<0.0001	<0.0001
bta04976	Bile secretion	30/1,021	96/7,748	<0.0001	0.0003
bta00052	Galactose metabolism	14/1,021	30/7,748	<0.0001	0.0006
bta04977	Vitamin digestion and absorption	13/1,021	27/7,748	<0.0001	0.0006
bta04978	Mineral absorption	21/1,021	60/7,748	<0.0001	0.0006
bta4146	Peroxisome	27/1,021	89/7,748	<0.0001	0.0007
bta01200	Carbon metabolism	34/1,021	126/7,748	<0.0001	0.0008
bta04530	Tight junction	41/1,021	166/7,748	<0.0001	0.0011
bta00511	Other glycan degradation	10/1,021	19/7,748	<0.0001	0.0012
bta00500	Starch and sucrose metabolism	13/1,021	30/7,748	<0.0001	0.0012
bta00830	Retinol metabolism	19/1,021	57/7,748	<0.0001	0.0017
bta4142	Lysosome	34/1,021	133/7,748	<0.0001	0.0017
bta04931	Insulin resistance	29/1,021	107/7,748	<0.0001	0.0017
bta00053	Ascorbate and aldarate metabolism	10/1,021	23/7,748	0.0003	0.0068
bta00510	N-Glycan biosynthesis	16/1,021	50/7,748	0.0004	0.0083
bta00280	Valine, leucine and isoleucine degradation	16/1,021	51/7,748	0.0005	0.0099
bta00561	Glycerolipid metabolism	18/1,021	61/7,748	0.0006	0.0099
bta03320	PPAR signaling pathway	21/1,021	79/7,748	0.0010	0.0159
bta00310	Lysine degradation	18/1,021	65/7,748	0.0013	0.0204
bta04974	Protein digestion and absorption	23/1,021	92/7,748	0.0014	0.0204
bta04152	AMPK signaling pathway	28/1,021	120/7,748	0.0014	0.0204
bta04975	Fat digestion and absorption	14/1,021	46/7,748	0.0017	0.0225
bta00640	Propanoate metabolism	11/1,021	34/7,748	0.0030	0.0391
bta05226	Gastric cancer	30/1,021	140/7,748	0.0040	0.0479

^1^GeneRatio = ratio of upregulated DEGs to all genes in a KEGG term; BgRatio = background ratio, ratio of all genes concerning this KEGG term to all genes in the background KEGG database; *pvalue* = probability value for hypergeometric test; *padj* = probability value for hypergeometric test adjusted for Benjamini-Hochberg FDR correction.

The KEGG pathways that tended to be upregulated (*padj* < 0.10) with exogenous GLP-2 administration were citrate cycle (TCA cycle), amino sugar and nucleotide sugar metabolism, tryptophan metabolism, arginine biosynthesis, serotonergic synapse, pentose and glucoronate interconversions, fructose and mannose metabolism, glycolysis/gluconeogenesis, Hippo signaling pathway, ether lipid metabolism, ABC transporters, drug metabolism–cytochrome P450, pyruvate metabolism, porphyrin and chlorophyll metabolism, and protein processing in endoplasmic reticulum (Table 3).

**Table 3:** Enriched KEGG pathways of the jejunal mucosa that tended to be upregulated with exogenous GLP-2 administration^1^.

KEGG ID	Description	GeneRatio	BgRatio	*pvalue*	*padj*
bta00020	Citrate cycle (TCA cycle)	10/1,021	31/7,748	0.0047	0.0516
bta00520	Amino sugar and nucleotide sugar metabolism	14/1,021	51/7,7788	0.0048	0.0516
bta00380	Tryptophan metabolism	13/1,021	46/7,788	0.0049	0.0516
bta00220	Arginine biosynthesis	8/1,021	22/7,788	0.0049	0.0516
bta04726	Serotonergic synapse	23/1,021	102/7,788	0.0057	0.0579
bta00040	Pentose and glucuronate interconversions	10/1,021	32/7,788	0.0060	0.0586
bta00051	Fructose and mannose metabolism	11/1,021	37/7,788	0.0062	0.0586
bta00010	Glycolysis/Gluconeogenesis	17/1,021	69/7,788	0.0066	0.0606
bta04390	Hippo signaling pathway	31/1,021	152/7,788	0.0073	0.0658
bta00565	Ether lipid metabolism	14/1,021	54/7,788	0.0082	0.0704
bta02010	ABC transporters	16/1,021	65/7,788	0.0083	0.0704
bta00982	Drug metabolism–cytochrome P450	14/1,021	56/7,788	0.0114	0.0905
bta00860	Pyruvate metabolism	11/1,021	40/7,788	0.0116	0.0905
bta00860	Porphyrin and chlorophyll metabolism	11/1,021	40/7,788	0.0116	0.0905
bta04141	Protein processing in endoplasmic reticulum	35/1,021	183/7,788	0.0127	0.0968

^1^GeneRatio = ratio of upregulated DEGs to all genes in a KEGG term; BgRatio = background ratio, ratio of all genes concerning this KEGG term to all genes in the background KEGG database; *pvalue* = probability value for hypergeometric test; *padj* = probability value for hypergeometric test adjusted for Benjamini-Hochberg FDR correction.

Downregulated KEGG pathways (*padj* < 0.05) in response to exogenous GLP-2 administration include ribosome, RNA transport, DNA replication, ribosome biogenesis in eukaryotes, spliceosome, cell cycle, base excision repair, mismatch repair, nucleotide excision repair, primary immunodeficiency, RNA polymerase, Fanconi anemia pathway, B cell receptor signaling pathway, and leishmaniasis (Table 4).

**Table 4:** Enriched KEGG pathways of the jejunal mucosa that were downregulated with exogenous GLP-2 administration^1^.

KEGG ID	Description	GeneRatio	BgRatio	*pvalue*	*padj*
bta03010	Ribosome	121/1,121	296/7,788	<0.0001	<0.0001
bta03013	RNA transport	72/1,121	187/7,788	<0.0001	<0.0001
bta03030	DNA replication	26/1,121	38/7,788	<0.0001	<0.0001
bta03008	Ribosome biogenesis in eukaryotes	35/1,121	76/7,788	<0.0001	<0.0001
bta03040	Spliceosome	56/1,121	170/7,788	<0.0001	<0.0001
bta04110	Cell cycle	47/1,121	143/7,788	<0.0001	<0.0001
bta03410	Base excision repair	18/1,121	33/7,788	<0.0001	<0.0001
bta03430	Mismatch repair	15/1,121	24/7,788	<0.0001	<0.0001
bta03420	Nucleotide excision repair	19/1,121	47/7,788	<0.0001	0.0004
bta05340	Primary immunodeficiency	20/1,121	60/7,788	0.0002	0.0051
bta03020	RNA polymerase	13/1,121	32/7,788	0.0003	0.0068
bta03460	Fanconi anemia pathway	18/1,121	53/7,788	0.0003	0.0068
bta04662	B cell receptor signaling pathway	29/1,121	109/7,788	0.0006	0.0133
bta05140	Leishmaniasis	28/1,121	106/7,788	0.0008	0.0173

^1^GeneRatio = ratio of downregulated DEGs to all genes in a KEGG term; BgRatio = background ratio, ratio of all genes concerning this KEGG term to all genes in the background KEGG database; *pvalue* = probability value for hypergeometric test; *padj* = probability value for hypergeometric test adjusted for Benjamini-Hochberg FDR correction.

### mRNA expression of jejunal carbohydrases, hexose transporters, and transcription factors

Exogenous GLP-2 administration did not influence (*padj* = 0.17) *SI* mRNA expression in the jejunum but tended to increase (*padj* = 0.07) jejunal *MGAM* mRNA expression (Table 5). Jejunal *LCT* and *TREH* mRNA expression were increased (*padj* < 0.05) with exogenous GLP-2 administration. Exogenous GLP-2 administration increased (*padj* = 0.01) the jejunal mRNA expression of *SLC5A1* and tended to increase (*padj* = 0.09) the jejunal mRNA expression of *SLC2A2*. Jejunal *SLC2A5* and *CDX2* mRNA expression were not influenced (*padj* > 0.15) by exogenous GLP-2 administration. Exogenous GLP-2 increased (*padj* < 0.01) or tended to increase (*padj* = 0.08) the mRNA expression of genes encoding the transcriptional coactivators, *HNF1A* and *GATA4*. Jejunal *KAT2B* mRNA expression was upregulated (*padj* = 0.01) by exogenous GLP-2 administration.

**Table 5:** Effects of exogenous GLP-2 administration on mRNA expression of genes encoding brush border carbohydrases, intestinal glucose transporters, and transcriptional regulators of carbohydrate-responsive genes^1^.

Ensembl ID	Name	Gene symbol	log2 fold change	*pvalue*	*padj*
ENSBTAG00000006975	Sucrase-isomaltase	*SI*	0.6363	0.0573	0.1789
ENSBTAG00000046152	Maltase-glucoamylase	*MGAM*	1.1820	0.0120	0.0732
ENSBTAG00000015170	Lactase	*LCT*	1.7571	0.0021	0.0287
ENSBTAG00000016202	Trehalase	*TREH*	0.7545	0.0065	0.0532
ENSBTAG00000012851	Solute carrier family 5 member 1	*SLC5A1*	1.4867	0.0004	0.0139
ENSBTAG00000005386	Solute carrier family 2 member 2	*SLC2A2*	1.1797	0.0178	0.0913
ENSBTAG00000034323	Solute carrier family 2 member 5	*SLC2A5*	1.7994	0.0618	0.1870
ENSBTAG00000001819	Caudal type homeobox 2	*CDX2*	0.3684	0.0468	0.1586
ENSBTAG00000021795	HNF1 homeobox A	*HNF1A*	0.9297	0.0001	0.0065
ENSBTAG00000005425	GATA binding protein 4	*GATA4*	0.5157	0.0147	0.0822
ENSBTAG00000006567	Lysine acetyltransferase 2B	*KAT2B*	0.5998	0.0002	0.0109

^1^*pvalue* = probability value for hypergeometric test; *padj* = probability value for hypergeometric test adjusted for Benjamini-Hochberg FDR correction.

## Discussion

### Influence of postruminal casein infusion on the jejunal transcriptome

To our knowledge, this is the first study that has evaluated the effects of postruminal casein infusion on the jejunal mucosal transcriptome in ruminants. Several plausible explanations exist as to why postruminal casein infusion for 7 d did not affect the jejunal transcriptome in the current study. The basal diet was formulated to exceed net energy and metabolizable protein requirements, whereas effects of postruminal casein infusion might have been detected if dietary energy or protein was limiting. For example, small intestinal starch disappearance increased by 37.6 percentage units (from 54.9% to 92.5%) when postruminal casein was added to a protein-free diet [55]. By comparison, postruminal casein infusion only increased small intestinal starch disappearance by a maximum of 10.2 percentage units when dietary metabolizable protein requirements were met [12]. Supporting this concept, a recent review summarized the effects of postruminal casein infusion from 51 studies and concluded that casein increased DM intake when metabolizable protein supply was deficient and that casein had no effect or decreased DM intake when metabolizable protein supply was in excess [56]. Therefore, it is possible that the lack of transcriptomic response to postruminal casein infusion was due to the positive energy and metabolizable protein balance of steers in the current study.

The method of nutrient administration, time to collect jejunal samples after infusion termination, and approach used to measure gene expression could have influenced the outcome of the current experiment. In the current study, casein was delivered continuously via postruminal infusion for 7 d. It is possible that the continuous postruminal infusion of casein masked effects that would be observed with a single pulse dose or multiple pulse doses per day. Previous research demonstrated that the timing of sampling in relation to luminal nutrient flow is important for detecting DEGs in the intestinal mucosa of cattle [57]. Li [57] found that the number of DEGs in duodenal mucosa decreased from 1490 DEGs to 482 DEGs 24 h after terminating the postruminal infusion of partially hydrolyzed starch. Small intestinal transit time is approximately 3 h in cattle [12] and all steers in the current study were slaughtered within a maximum of 3 to 4 h after termination of the 7-d postruminal infusion. Therefore, it is possible that the final portion of luminal casein had passed the jejunum by the time samples were collected for analysis. Because the intestinal mucosa is a heterogeneous tissue with numerous cell types [24], approaches using single-cell RNA sequencing technology could potentially detect DEGs of lowly expressed cell populations, such as enteroendocrine cells, that were likely underrepresented in the current study [58].

A common finding between the current study and Li [57] is that 7-d postruminal infusion of partially hydrolyzed starch or raw corn starch with casein does not influence many functions of the intestinal epithelium. Although previous research detected 1490 DEGs, the enrichment analyses showed that DEGs found after 7-d of postruminal partially hydrolyzed starch infusion only affected a few, generic gene ontologies of the duodenal mucosa (such as biological process, cellular process, molecular function, binding) [57]. Likewise, postruminal infusion of casein did not result in any enriched KEGG pathways or gene ontologies in the jejunal mucosa in the current study. These findings collectively suggest that continuous postruminal infusion of starch or starch with casein for 7 d does not influence the transcriptome of the intestinal mucosa in cattle.

Increased small intestinal starch disappearance, pancreatic mass, and α-amylase activity have been consistent responses to postruminal casein infusion in ruminants [5–15, 17, 23, 55, 59]. Notably, the lack of jejunal transcriptomic response in the current study indicates that increasing pancreatic α-amylase activity via 7-d postruminal casein infusion does not increase the mRNA expression of jejunal brush border carbohydrases. These findings support the previously proposed concept that carbohydrases of the bovine pancreas and small intestinal mucosa do not coordinate a response to luminal nutrient flows [23]. Factors mediating the positive response of increased small intestinal starch digestion to postruminal casein may include increasing pancreatic α-amylase activity [23], increasing the digestion of other nutrients [7, 13, 16], stimulating microbial activity [60–62], changing passage rate [62], or fostering chemical interactions with starch in the small intestinal lumen [63, 64].

### Effects of exogenous GLP-2 on transcriptional mechanisms associated with jejunal mucosal growth

**Fig 4:**
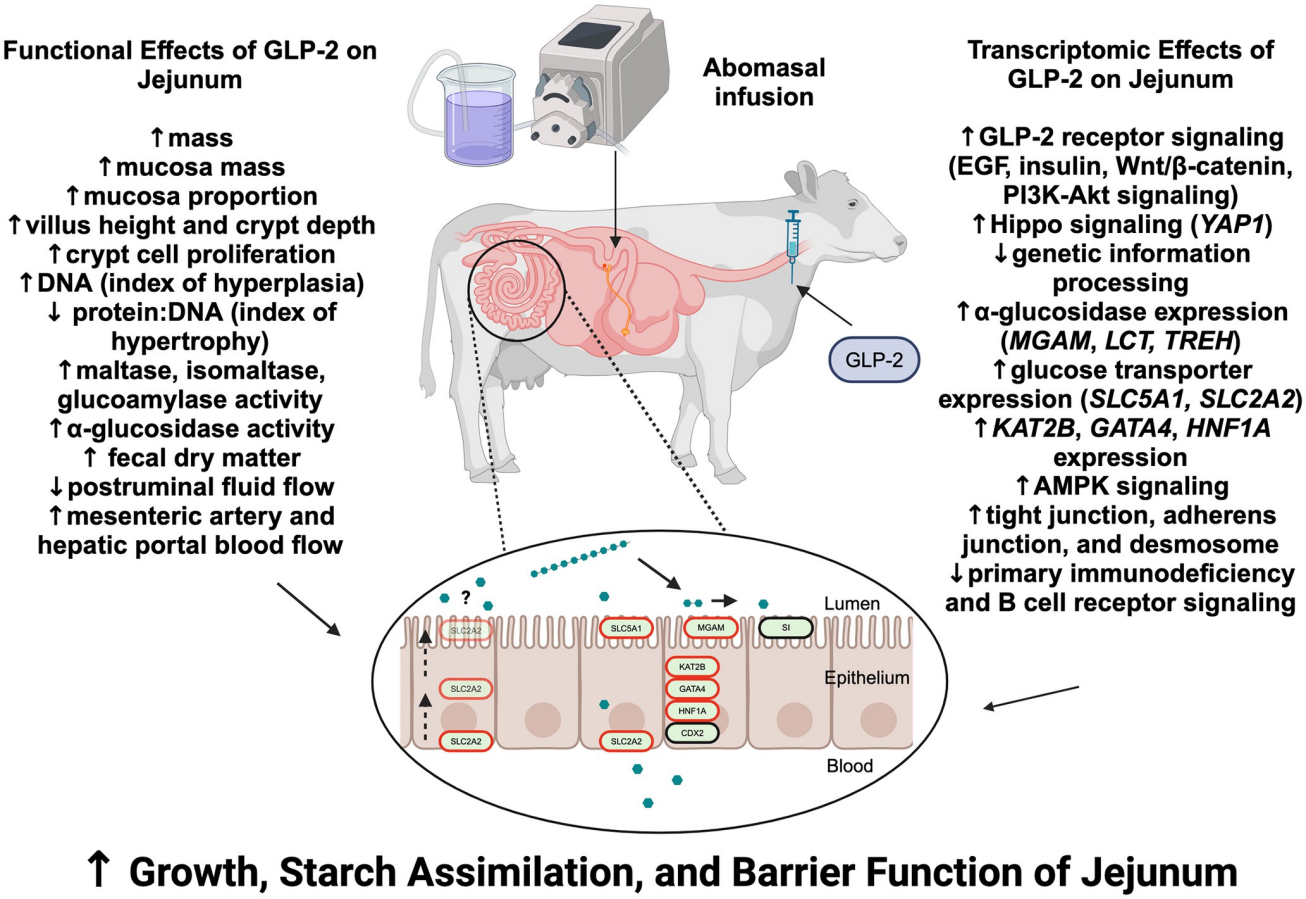
Schematic of the selected functional and transcriptomic effects of exogenous GLP-2 administration on jejunal mucosal growth and small intestinal starch assimilation capacity. Functional effects were summarized from studies in cattle [23, 32]. Transcriptomic effects were summarized from the current study. Figure was created with BioRender.com.

A schematic of the functional and transcriptomic effects of exogenous GLP-2 on the jejunum in cattle is summarized (Fig 4). Exogenous GLP-2 administration for 7 d increased jejunal mucosal mass, mucosal DNA content, and mucosal protein content of steers used in the current study by 45.6%, 32.6%, and 42.8%, respectively [23]. Exogenous GLP-2 administration also increases jejunal villus height, crypt depth, and crypt cell proliferation in cattle [32]. After GLP-2 binds to its receptor, it is generally thought that independent actions of insulin-like growth factor 1 (IGF-1) and epidermal growth factor (EGF) are critical downstream mediators of increased cellular proliferation [65–68]. These mediators are thought to lead to activation of Wnt/β-catenin and PI3K/Akt signaling pathways to increase cellular proliferation and differentiation [65, 69]. Currently, there are no annotations of GLP-2 receptor signaling pathways in the KEGG pathway or gene ontology databases. Nevertheless, several transcripts associated with EGF receptor signaling (*ERBB2*, *ERBB3*), insulin signaling (*INSR*, *IRS2*), Wnt/β-catenin signaling (*WNT1*, *WNT8B*, *WNT11*, *FZD3*, *FZD5*, *DVL1*, *GSK3B*, *CTNNB1*, *TCF7L2*) and PI3K/Akt signaling (*PDPK1*) pathways were upregulated with exogenous GLP-2 administration. These data suggest that part of the increase in jejunal mucosal growth of steers exposed to exogenous GLP-2 in the current study might have occurred through the activation of GLP-2 receptor signaling pathways.

Environmental and cellular cues between conserved signaling pathways (Wnt/β-catenin, TGF-β, Notch, Hedgehog) can lead to cross-talk influencing the activation of the Hippo signaling pathway [70]. The Hippo signaling pathway tended to be upregulated in response to exogenous GLP-2 administration in the current study. In addition to the upregulated transcripts associated with cross-talk from Wnt/β-catenin signaling, key transcriptional regulators of the Hippo signaling pathway (*MOB1B*, *YAP1*, *SMAD3*, *SMAD9*) controlling cellular proliferation and apoptosis were upregulated in response to exogenous GLP-2 administration. Protein kinases of the Hippo signaling pathway can be regulated by several upstream inputs from intracellular, extracellular, and noncellular components including G protein-coupled receptors, metabolism, mechanical stimuli, cell polarity and density, and stress [70, 71]. Notably, upregulation of transcripts encoding for cadherin 1 (*CDH1*), an adherens junction protein, and its mammalian ajuba family member (*LIMD1*) suggest that protein kinase activity (MST1/2, LATS1/2) regulating the Hippo signaling pathway was inhibited, allowing YAP1 translocation to the nucleus to function as a transcriptional co-activator of pro-proliferation and anti-apoptosis genes [72–74].

Net tissue accretion can be due to a combination of increasing cellular proliferation and survival and decreasing cellular apoptosis and proteolysis [19, 21, 32, 35, 75–77] and activation of these pathways by GLP-2 could vary based on physiological state, the presence of luminal nutrient supply, and with mucosal inflammation/injury [19–21]. Because increased mucosal mass can occur via multiple mechanisms of increasing proliferation and decreasing apoptosis, there can be multiple mediators of GLP-2-induced mucosal growth [65]. In the current study, many KEGG pathways associated with genetic information processing were downregulated with exogenous GLP-2 administration including ribosome, RNA transport, DNA replication, ribosome biogenesis in eukaryotes, spliceosome, cell cycle, base excision repair, mismatch repair, nucleotide excision repair, and RNA polymerase. In addition, several transcripts associated with ubiquitin-mediated proteolysis (30/166) were downregulated with exogenous GLP-2 administration, which suggests that part of the increase in jejunal mucosal growth with exogenous GLP-2 administration occurred through decreased proteolysis in the current study. Steers received subcutaneous GLP-2 injections twice daily for 7 d in the current study, and tissues were collected on day 8. Therefore, jejunal mucosa was collected approximately 14–15 h after the last GLP-2 injection was given on day 7. Glucagon-like peptide 2 has a short half-life (7 min) in plasma in humans [78] but, subcutaneous GLP-2 administration can sustain elevated pharmacological plasma GLP-2 concentrations for more than 7 h in cattle [32]. It is possible that changes in the mRNA expression of genes in genetic information processing pathways are transient and that desensitization of the GLP-2 receptor could have occurred with chronic GLP-2 treatment for 7 d [32]. It is possible that the effects of exogenous GLP-2 for 7 d on genetic information processing pathways occurred because of decreased cellular apoptosis and proteolysis, chronic exposure causing GLP-2 receptor desensitization, timing of sampling, or because the pharmacological dose given could potentially differ from responses that would occur under normal physiological conditions.

### Effects of exogenous GLP-2 on transcriptional mechanisms associated with jejunal starch assimilation

Epithelial cells are the predominant cell types (> 80%) of the jejunal mucosa [79] and the brush border membrane is estimated to contain approximately 10% to 11% of the total protein content of epithelial cells [80]. Sucrase-isomaltase and maltase-glucoamylase comprise 8.2% and 2.7% of the total protein content of the brush border membrane in humans [81]. Therefore, increasing the growth of the jejunal mucosa via the administration of intestinotrophic hormones, like GLP-2, could potentially result in increased expression of genes encoding brush border membrane proteins [82] (hydrolases, channels and transporters, cytoskeletal components, myosin motor proteins, and adhesion proteins) that are important for adaptation of the intestinal mucosa to high-grain diets [24]. In addition to the brush border of absorptive epithelial cells, the jejunal mucosa sampled in the current study for high-throughput RNA sequencing contains multiple cell types (goblet cells, immune cells, and enteroendocrine cells) that could have specialized functions in response to changes in luminal nutrient flows and exogenous GLP-2 administration [24].

Several prior studies spanning different animal models have demonstrated that exogenous GLP-2 can increase the mRNA expression and enzyme activity of brush border carbohydrases [22, 23, 76, 83, 84]. Brubaker [76] was the first to demonstrate that exogenous GLP-2 increased duodenal maltase activity in mice, but did not influence sucrase or lactase activities. In parenterally-fed rats, intravenous administration of GLP-2 increased jejunal and ileal sucrase-isomaltase mRNA expression [83]. Exogenous GLP-2 administration has been shown to increase sucrase-isomaltase and maltase-glucoamylase mRNA expression and activity in pre-term parenterally-fed neonatal piglets [22]. However, effects of exogenous GLP-2 on sucrase-isomaltase mRNA expression and activity were not observed when administered to full-term parenterally-fed neonatal piglets [84] and enteral nutrition decreased sucrase, maltase, and lactase activities compared with parenteral nutrition [22]. These studies indicate that effects of exogenous GLP-2 on brush border carbohydrase expression and activity are influenced by species, age, and luminal nutrient flow.

We demonstrated that exogenous GLP-2 administration increased jejunal maltase, isomaltase, and glucoamylase activities in the current study [23]. In accordance with increased jejunal α-glucosidase activity, genes associated with brush-border carbohydrate digestion including maltase-glucoamylase, lactase, and trehalase (*MGAM*, *LCT*, *TREH*) were upregulated or tended to be upregulated in the jejunal mucosa of steers treated with exogenous GLP-2 in the current study. It had been suggested that GLP-2-induced suppression of proteolysis and apoptosis may contribute to increased *MGAM* expression and activity but, more specific mechanisms may be involved [22]. Caudal type homeobox 2 (*CDX2*) has long been thought to be an important transcription factor contributing to the upregulation of *MGAM* [22, 83]. However, other authors did not find a strong association between *CDX2* and *MGAM* mRNA expression in their studies [22, 83] and we did not find evidence of this association in our current study, as the mRNA expression of *CDX2* was not influenced by exogenous GLP-2 administration. Recent experiments have demonstrated that carbohydrate-responsive genes (*SI*, *MGAM*, *SLC5A1*) are transcriptionally regulated by histone H3 modifications at lysine residues (acetylation/methylation) and increased coactivator binding (KAT2B, CDX2, HNF1, GATA4) in the promoter/enhancer and transcribed regions of those genes in rodents fed high-starch diets [85–89]. In accordance with these findings in rodents, we found that genes associated with transcriptional regulation of *MGAM*, *SI*, and *SLC5A1* (*KAT2B*, *HNF1*, *GATA4*) were upregulated by exogenous GLP-2 administration. Our data suggests that exogenous GLP-2 administration increased *MGAM* transcription by increasing histone H3 acetylation and increasing interactions of transcriptional coactivators in the promoter and transcribed mRNA regions [90].

Of note, exogenous GLP-2 administration did not increase jejunal *SI* mRNA expression in the current study. In humans and mice, approximately 80% of the apparent maltase activity is derived from sucrase-isomaltase and the remaining 20% is derived from maltase-glucoamylase [91, 92]. In humans, maltase-glucoamylase is the primary protein exhibiting α-glucosidase activity at low substrate concentrations while sucrase-isomaltase is the primary protein exhibiting α-glucosidase activity at high substrate concentrations [93]. Others have suggested that the opposite response may occur in ruminants, because of the lower *K*_*m*_ value for isomaltase compared to maltase in jejunal homogenates from cattle [94]. The upregulation of jejunal *MGAM* mRNA expression and activity in the current study may support this concept. Although ruminants do not contain sucrase activity [37], it should be acknowledged that small intestinal *SI* mRNA expression can be transcriptionally regulated by luminal nutrient flows in cattle, as increasing dietary fructose consumption in milk replacer decreased small intestinal *SI* mRNA expression in calves [95]. The absence of intestinal sucrase activity [96], missing stalk region of sucrase-isomaltase [37], lack of transcriptional response of *SI* to exogenous GLP-2, and lack of effect of GLP-2 on postruminal starch disappearance [23] leaves many questions about the regulation of α-glucosidase activity in ruminants to be answered. Limitations in the extent of small intestinal starch digestion in ruminants might be similar to humans with phenotype V of congenital sucrase-isomaltase deficiency [37] and may not be completely overcome by exogenous GLP-2 administration.

Exogenous GLP-2 administration has been shown to increase the mRNA expression, protein abundance, and activity of intestinal glucose transporters in rodents [97–100] and net portal glucose absorption in parenterally-fed piglets [101, 102]. A prior study had demonstrated that intravenous GLP-2 infusion increased hepatic portal plasma flow but, did not influence net portal glucose absorption in cattle fed a 50:50 alfalfa hay and calf starter diet and fasted for 12 h prior to measurements [103]. To date, the effects of exogenous GLP-2 administration on portal glucose absorption in fed animals has not been evaluated. Although portal glucose absorption was not measured in the current study, we found that exogenous GLP-2 administration increased or tended to increase the expression of genes encoding SGLT1 (*SLC5A1*) and GLUT2 (*SLC2A2*) in cattle postruminally infused with starch, suggesting that the capacity for apical and basolateral glucose transport might have increased with exogenous GLP-2 administration. Like *MGAM*, *SLC5A1* mRNA expression is transcriptionally regulated by histone H3 acetylation and co-activator binding (*HNF1*, *GATA4*, and *CDX2*) in both the promoter and transcribed regions [87–89, 104–107] and we found evidence of these associations in the current study. It has also been demonstrated that activation of the sweet taste receptor complex (T1R2-T1R3) of enteroendocrine L-cells with artificial sweeteners results in increased villus height and crypt depth, GLP-2 secretion, maltase activity, SGLT1 protein abundance, and glucose uptake activity in small intestinal tissue from ruminants [108]. Further investigations using GLP-2 receptor knockout mice have revealed that GLP-2-induced increases in SGLT1 expression and glucose uptake activity are dependent on GLP-2 receptor binding [109]. After GLP-2 binds to its receptor, vasoactive intestinal peptide or pituitary adenylate cyclase-activating polypeptide bind to VIPR1, increasing intracellular cAMP, resulting in increased SGLT1 expression [109]. Notably, we found that exogenous GLP-2 administration increased jejunal *VIPR1* mRNA expression in the current study, which may indicate a role of vasoactive intestinal peptide in the downstream regulation of *SLC5A1* mRNA expression by GLP-2 in cattle.

Interestingly, exogenous GLP-2 administration has been shown to promote the translocation of GLUT2 from the basolateral membrane to the apical membrane in the rat jejunum to provide a low affinity, high capacity hexose transport mechanism when luminal hexose concentrations exceed transport capacity by the high affinity, low capacity glucose transporter, SGLT1 [100]. The apical GLUT2 translocation hypothesis [110] is highly controversial [111, 112] largely due to the methodology used to assess its validity under normal physiological conditions and because of contamination that occurs during the isolation of brush border membranes. Some evidence for GLUT2 localization on the apical membrane in lactating dairy cows and neonatal calves exists [113–115]. In the current study, it is difficult to speculate whether a tendency to increase *SLC2A2* mRNA expression resulted in greater GLUT2 protein abundance or activity or the amount of GLUT2 protein localized to each membrane. Differentially expressed genes associated with positive regulation of protein exit from the endoplasmic reticulum and localization to the plasma membrane were significantly enriched with exogenous GLP-2 administration. Sphingolipids contribute to lipid raft formation and intracellular translocation which aid in the trafficking of proteins to the membrane and numerous gene ontology terms related to sphingolipid metabolism and signaling were upregulated with exogenous GLP-2 administration. In the current study, exogenous GLP-2 upregulated the AMPK signaling KEGG pathway, and its activation has been associated with GLUT2 insertion into the apical membrane [116, 117]. In contrast, the expression of genes encoding proteins associated with suspected mechanisms of apical GLUT2 translocation (*CACNA1D*, *TAS1R2*, *TAS1R3*, *PRKCB*) [117–120] were not upregulated by exogenous GLP-2 administration in the current study. Conflicting points of evidence from the current study provide justification for further investigation of the apical GLUT2 translocation hypothesis in cattle.

Tissues of the portal-drained viscera, including the small intestine, utilize large amounts of glucose to maintain metabolic homeostasis [39, 121–123]. Increasing luminal starch flow in the small intestine results in increased glucose utilization by the portal-drained viscera [124]. With this knowledge from prior studies and the likely increases in α-glucoside hydrolysis and intestinal glucose uptake with exogenous GLP-2 administration in the current study, it would be expected that exogenous GLP-2 administration would increase glucose utilization by the jejunal mucosa. As expected, exogenous GLP-2 upregulated several genes associated with starch and sucrose metabolism and galactose metabolism KEGG pathways. In addition, exogenous GLP-2 upregulated several gene ontologies related to carbohydrate digestion, absorption, and metabolism including carbohydrate metabolic process, hydrolase activity, glucosidase activity, monosaccharide metabolic process, and hexose metabolic process. Glycolysis/gluconeogenesis (*HKDC1*, *HK2*, *PGM1*, *PCK2*, *PDHA1*) and the tricarboxylic acid cycle (*ACO1*, *IDH1*, *SDHA*, *PC*, *DLD*, *SUCLG1*, *SUCLG2*) KEGG pathways tended to upregulate with exogenous GLP-2 administration. These findings suggest that glucose utilization by the jejunal mucosa increased with exogenous GLP-2 administration. Overall, the current study provides a transcriptional overview of the mechanisms associated with increased jejunal starch assimilation capacity in response to exogenous GLP-2 administration.

### Effects of exogenous GLP-2 on transcriptional mechanisms associated with intestinal barrier function

In addition to nutrient digestion and absorption, the gastrointestinal tract acts as a barrier, limiting permeability to potentially toxic or pathogenic organisms and compounds. Epithelial permeability of the intestinal mucosa can occur through transcellular transport associated with solute or water transport through a cell or paracellular transport in between cell-cell junctions [125]. In our study, exogenous GLP-2 administration tended to decrease postruminal fluid flow and increase fecal dry matter content, suggesting increased postruminal fluid absorption [23]. Exogenous GLP-2 administration has been shown to decrease jejunal conductance and *ex vivo* fluxes of Na^+^, CrEDTA, and horseradish peroxidase in mice [126]. Cameron and Perdue [127] found that exogenous GLP-2 administration decreased bacteria adhering to and penetrating the intestinal mucosa in stress-induced mice.

Others have found that exogenous GLP-2 or GLP-2 receptor agonist administration increased the mRNA expression of genes encoding tight junction proteins [128–131]. In the current study, exogenous GLP-2 administration upregulated adherens junction and tight junction KEGG pathways. In these KEGG pathways, 36% and 25% of the total number of transcripts in each KEGG pathway term were upregulated, respectively. Several gene ontologies including transcripts encoding tight junction proteins were upregulated with exogenous GLP-2 administration including apical junction complex, apical, basolateral, and lateral plasma membrane; bicellular tight, cell-cell, occluding junction; cell, cell-cell junction organization; epithelial cell differentiation and morphogenesis; apical junction, cell, cell-cell, and bicellular tight junction assembly; and anion and chloride transport. Also, connections of adherens and tight junction proteins to the actin cytoskeleton through adaptor proteins may have been improved, as indicated by an upregulated cluster of actin-based cell projections, anchoring junction, actin-based cell projections, actin cytoskeleton, and desmosome gene ontologies. Upregulation of gene ontologies related to phospholipid and sphingolipid metabolism and terms related to apical membrane suggest that exogenous GLP-2 increased the physical barrier (plasma membrane) of individual cells of the jejunal mucosa. These data suggest that exogenous GLP-2 administration enhanced the physical barrier function of the jejunal mucosa by decreasing transcellular (plasma membrane) and paracellular (tight and adherens junction) permeability. Increased postruminal fluid absorption coupled with increased mRNA expression of cell-cell junction proteins suggest that the capacity for absorption was increased and that selective permeability of the jejunal epithelium was improved.

In addition to the physical barrier of the small intestine, intestinal permeability can be affected by exogenous factors, epithelial cell turnover, cytokines, and immune cells [125]. Notably, intestinal barrier dysfunction is associated with increased intestinal inflammation and initiation of immunoregulatory processes [132]. In the current study, exogenous GLP-2 administration downregulated primary immunodeficiency and B cell receptor signaling KEGG pathways. Gene ontologies related to immune function that were downregulated with exogenous GLP-2 administration include B cell activation, regulation of B cell activation, immunoglobulin-mediated immune response, B cell-mediated immunity, immunoglobulin production, regulation of lymphocyte activation, adaptive immune response, and others. These data suggest that the adaptive immune function of the jejunal mucosa was improved with exogenous GLP-2 administration. Collectively, next-generation RNA sequencing findings of the current study suggest that increased jejunal mucosal growth with exogenous GLP-2 administration may be associated with improved intestinal barrier function, which was supported by changes in KEGG pathways and gene ontologies associated with decreased intestinal permeability, inflammation, and immune system activation.

### Implications for animal growth and feed efficiency

To our knowledge, this is the first report that has investigated the effects of exogenous GLP-2 on the jejunal mucosal transcriptome using an untargeted RNA-sequencing approach in mammals. Although our primary focus was to describe transcriptional mechanisms affected by exogenous GLP-2 and its relationship with phenotypic responses (growth, starch assimilation, barrier function), this dataset could be useful for identifying novel functions of exogenous GLP-2 and lead to future investigations detailing mechanisms of GLP-2-mediated responses in the small intestinal mucosa. Comparing the findings of the current study with other RNA-sequencing datasets shows that DEGs and KEGG pathways affected by exogenous GLP-2 are similar to DEGs and KEGG pathways identified in cattle with divergent average daily gain [133, 134]. Notably, the mRNA expression of *APOB*, *CLCA4*, *CUBN*, *CYP2B6*, and *SLC9A3* were differentially expressed in the jejunal mucosa in all 3 studies. Cattle with greater average daily gain exhibit 9 upregulated KEGG pathways of the jejunal mucosa [133] that were also found to be upregulated in the current study in response to exogenous GLP-2 administration. These pathways include vitamin digestion and absorption, galactose metabolism, mineral absorption, bile secretion, retinol metabolism, starch and sucrose metabolism, protein digestion and absorption, PPAR signaling pathway, and insulin resistance.

It has been suggested that increasing proliferation of the small intestinal epithelium could result in an increased capacity for nutrient absorption that would typically outweigh increased energy expenditure for maintaining additional tissue [24]. Fitzsimons [135] hypothesized that beef cattle with greater gain:feed have a greater capacity for nutrient absorption from the small intestine. Supporting this hypothesis, others have found that jejunal mucosal density is positively correlated with gain:feed [136]. Also, the number of cells in the duodenal and ileal mucosa is positively correlated with improvements in feed efficiency [137]. Those authors suggested that the benefits of increased metabolic activity in the small intestinal mucosa due to increased cellularity would be greater than the increased energetic cost of maintaining the tissue, leading to improved feed efficiency [137]. Our phenotypic [23] and transcriptomic data from the current study suggest that some of the metabolic pathways and molecular functions of the jejunal mucosa affected by exogenous GLP-2 administration may also underlie physiological differences in feed efficiency in beef cattle. These observations warrant future research to investigate if potential changes to small intestinal function with exogenous GLP-2 administration could lead to improved growth, health, and/or feed efficiency of beef cattle.

## Conclusions

Under the conditions of the current experiment, postruminal casein infusion for 7 d downregulated 7 genes of the jejunal mucosa but did not result in enriched KEGG pathways or gene ontologies. These findings might suggest that positive nutritional responses to postruminal casein infusion are mediated through alternative mechanisms or tissues. Results from the current study demonstrated that exogenous GLP-2 administration affected the expression of protein-coding genes that are associated with functions of the jejunal mucosa including mucosal growth, small intestinal starch assimilation, and intestinal barrier function. Differentially enriched pathways that were affected by exogenous GLP-2 administration may be related to phenotypes of improved nutrient assimilation capacity, animal growth, and feed efficiency. Next-generation RNA sequencing generated novel targets for future research to elucidate mechanisms of GLP-2-mediated responses in the jejunal mucosa.

## Supporting information

S1 File Upregulated DEG in response to exogenous GLP-2 administration. (XLSX)

S2 File Downregulated DEG in response to exogenous GLP-2 administration. (XLSX)

S3 File Upregulated DEG in response to postruminal casein infusion. (XLSX)

S4 File Downregulated DEG in response to postruminal casein infusion. (XLSX)

S5 File Upregulated gene ontology in response to exogenous GLP-2 administration. (XLSX)

S6 File Downregulated gene ontology in response to exogenous GLP-2 administration. (XLSX)

S7 File Upregulated KEGG pathway in response to exogenous GLP-2 administration. (XLSX)

S8 File Downregulated KEGG pathway in response to exogenous GLP-2 administration. (XLSX)
